# Supplementary material for: Identifying prognostic factors for survival in intensive care unit patients with SIRS or sepsis by machine learning analysis on electronic health records
Source: PLOS Digit Health. 2024 Mar 15;3(3):e0000459. doi: 10.1371/journal.pdig.0000459 (PMC10942078; doi:10.1371/journal.pdig.0000459)
Supplement: S1 Appendix — Text A: Formulas of the confusion matrix rates. Text B: Model Hyperparameters. Text C: Model performances. Text D: Model Calibration. Text E: Single Feature Elimination. Text F: Sensitivity Analysis: Hyperparameter Optimization. (PDF) [file pdig.0000459.s001.pdf]

S1 Appendix

Text A Formulas of the confusion matrix rates Here is the equation of the Matthews correlation coefficient (MCC) [38–41] and of the other rates to evaluate the confusion matrices considering 0.5 as probability threshold:

$$\text{MCC} \; = \frac{TP \cdot TN - FP \cdot FN}{\sqrt{(TP+FP)\cdot(TP+FN)\cdot(TN+FP)\cdot(TN+FN)}} \tag{1}$$

(minimum and worst value = −1; maximum and best value = +1)

$$\text{accuracy} = \frac{TP+TN}{TN+TP+FP+FN} \tag{2}$$

(minimum and worst value = 0; maximum and best value = 1)

$$\text{true positive rate, recall, sensitivity, TPR} = \frac{TP}{TP+FN} \tag{3}$$

(minimum and worst value = 0; maximum and best value = 1)

$$\text{true negative rate, specificity, TNR} = \frac{TN}{TN+FP} \tag{4}$$

(minimum and worst value = 0; maximum and best value = 1)

$$\text{F1-score} = \frac{2TP}{2TP+FN+FP} \tag{5}$$

(minimum and worst value = 0; maximum and best value = 1)

$$\text{positive predictive value, precision, PPV} = \frac{TP}{TP+FP} \tag{6}$$

(minimum and worst value = 0; maximum and best value = 1)

$$\text{negative predictive value, NPV} = \frac{TN}{TN+FN} \tag{7}$$

(minimum and worst value = 0; maximum and best value = 1)

$$\text{Precision-Recall (PR) curve} = \begin{cases} true\ positive\ rate & on\ the\ x\ axis \\ positive\ predictive\ value & on\ the\ y\ axis \end{cases} \tag{8}$$

(minimum and worst value = 0; maximum and best value = 1)

$$\text{ROC curve} = \begin{cases} false\ positive\ rate & on\ the\ x\ axis \\ true\ positive\ rate & on\ the\ y\ axis \end{cases} \tag{9}$$

(minimum and worst value = 0; maximum and best value = 1)

Text B Model Hyperparameters

Here the default hyperparameters of the trained models are listed:

- SVM: $kernel = "linear"$, $probability = "True"$, $class\_weight = "balanced"$, $C = 1.0$, $gamma = "scale"$, $shrinking = "True"$, $tol = 0.001$
- LR: $penalty = "l2"$, $class\_weight = "balanced"$, $tol = 0.0001$, $C = 1.0$, $fit\_intercept = "True"$, $intercept\_scaling = 1$
- *Tree*: $criterion = "gini"$, $splitter = "best"$, $max\_depth = None$, $min\_samples\_split = 2$, $min\_samples\_leaf = 1$, $min\_weight\_fraction\_leaf = 0.0$, $class\_weight = "balanced"$
- RF: $n\_estimators = 100$, $criterion = "gini"$, $max\_depth = None$, $min\_samples\_split = 2$, $min\_samples\_leaf = 1$, $min\_weight\_fraction\_leaf = 0.0$, $max\_features = "sqrt"$, $max\_leaf\_nodes = None$, $min\_impurity\_decrease = 0.0$, $bootstrap = "True"$, $class\_weight = "balanced"$
- XGB: $base\_score = 0.5$, $eval\_metric = "logloss"$, $colsample\_bylevel = 1$, $colsample\_bytree = 1$, $gamma = 0$, $max\_delta\_step = 0$, $min\_child\_weight = 1$, $missing = 1$, $nthread = -1$, $objective = "binary : logistic"$, $reg\_alpha = 0$, $reg\_lambda = 1$, $scale\_pos\_weight = 1/weight\_ratio$

Text C Model performances

MCC	***SEPSIS***	***SIRS***
SVM	**0.533**(0.456-0.574)	0.378(0.31-0.436)
LR	**0.533**(0.456-**0.591**)	0.379(0.313-0.451)
Tree	0.368(0.312-0.446)	0.289(0.202-0.378)
RF	0.516(0.431-0.573)	0.39(0.3-0.505)
XGB	0.459(0.397-0.534)	**0.489**(0.343-0.582)

Table A Median (first-third quartiles) of Matthews correlation coefficient (MCC) (Equation 1) obtained from five different machine learning model families for the *SEPSIS* and *SIRS* cohorts.

ACC	***SEPSIS***	***SIRS***
SVM	0.795(0.761-0.818)	0.908(0.896-0.926)
LR	0.807(0.761-0.83)	0.914(0.902-0.933)
Tree	0.75(0.727-0.784)	0.942(0.931-0.951)
RF	0.807(0.784-0.841)	0.963(0.957-0.969)
XGB	0.795(0.773-0.818)	0.963(0.957-0.969)

Table B Median (first-third quartiles) of Accuracy (ACC) (Equation 2) obtained from five different machine learning model families for the *SEPSIS* and *SIRS* cohorts.

SE	***SEPSIS***	***SIRS***
SVM	0.806(0.787-0.836)	0.918(0.899-0.93)
LR	0.813(0.776-0.836)	0.918(0.903-0.93)
Tree	0.806(0.776-0.851)	0.959(0.943-0.968)
RF	0.851(0.821-0.881)	0.981(0.968-0.987)
XGB	0.851(0.821-0.881)	0.975(0.968-0.981)

Table C Median (first-third quartiles) of Sensitivity (SE) (Equation 3) obtained from five different machine learning model families for the *SEPSIS* and *SIRS* cohorts.

SP	***SEPSIS***	***SIRS***
SVM	0.762(0.714-0.81)	0.8(0.6-0.8)
LR	0.786(0.714-0.81)	0.8(0.6-0.85)
Tree	0.571(0.524-0.667)	0.4(0.2-0.6)
RF	0.667(0.619-0.762)	0.4(0.4-0.6)
XGB	0.619(0.571-0.714)	0.6(0.4-0.65)

Table D Median (first-third quartiles) of Specificity (SP) (Equation 4) obtained from five different machine learning model families for the *SEPSIS* and *SIRS* cohorts.

F1	***SEPSIS***	***SIRS***
SVM	0.861(0.835-0.877)	0.951(0.944-0.961)
LR	0.865(0.834-0.882)	0.954(0.947-0.964)
Tree	0.833(0.812-0.853)	0.97(0.963-0.975)
RF	0.874(0.857-0.892)	0.981(0.978-0.984)
XGB	0.86(0.843-0.881)	0.981(0.977-0.984)

Table E Median (first-third quartiles) of F1-score (F1) (Equation 5) obtained from five different machine learning model families for the *SEPSIS* and *SIRS* cohorts.

PPV	***SEPSIS***	***SIRS***
SVM	0.918(0.9-0.935)	0.993(0.987-0.993)
LR	0.918(0.903-0.935)	0.993(0.986-0.995)
Tree	0.862(0.84-0.881)	0.981(0.975-0.987)
RF	0.896(0.871-0.917)	0.981(0.98-0.987)
XGB	0.881(0.862-0.905)	0.987(0.981-0.989)

Table F Median (first-third quartiles) of Positive Predictive Value (PPV) (Equation 6) obtained from five different machine learning model families for the *SEPSIS* and *SIRS* cohorts.

NPV	***SEPSIS***	***SIRS***
SVM	0.559(0.5-0.6)	0.217(0.186-0.25)
LR	0.569(0.5-0.607)	0.225(0.19-0.25)
Tree	0.483(0.443-0.539)	0.25(0.167-0.309)
RF	0.592(0.54-0.652)	0.4(0.286-0.5)
XGB	0.558(0.521-0.619)	0.429(0.333-0.5)

Table G Median (first-third quartiles) of Negative Predictive Value (NPV) (Equation 7) obtained from five different machine learning model families for the *SEPSIS* and *SIRS* cohorts.

AUPRC	***SEPSIS***	***SIRS***
SVM	0.956(0.939-0.965)	0.996(0.99-0.999)
LR	0.954(0.939-0.964)	0.995(0.99-0.999)
Tree	0.907(0.898-0.92)	0.99(0.987-0.993)
RF	0.95(0.936-0.962)	0.998(0.994-0.999)
XGB	0.94(0.928-0.952)	0.998(0.992-0.999)

Table H Median (first-third quartiles) of Area Under Precision-Recall Curve (AUPRC) (Equation 8) obtained from five different machine learning model families for the *SEPSIS* and *SIRS* cohorts.

AUROC	***SEPSIS***	***SIRS***
SVM	0.87(0.836-0.895)	0.904(0.82-0.966)
LR	0.865(0.836-0.888)	0.882(0.821-0.969)
Tree	0.694(0.661-0.734)	0.681(0.591-0.772)
RF	0.863(0.828-0.889)	0.936(0.849-0.968)
XGB	0.83(0.804-0.863)	0.93(0.846-0.97)

Table I Median (first-third quartiles) of Area Under Receiver Operating Curve (AUROC) (Equation 9) obtained from five different machine learning model families for the *SEPSIS* and *SIRS* cohorts.

Text D Model Calibration

S1 Fig reports the average calibration curves across the MCCV runs for the best and worst cases for both SIRS and SEPSIS populations. Here, it can be observed that despite the curves are not optimal on average, the best models show improved calibration with respect to the worst ones.

S1 Fig. Average calibration curves across the MCCV runs for the best (left column) and worst (right column) for SEPSIS (upper row) and SIRS (lower row).

Text E Single Feature Elimination Results of the single feature elimination procedure to assess feature inmportance.

	SEPSIS	***SIRS***
	LR	***XGB***
Age	0.533(0.475-0.58)	0.489(0.313-0.548)
APACHE II	**0.436(0.383-0.496)**	0.432(0.343-0.532)
CRP	0.528(0.462-0.591)	0.454(0.343-0.536)
EOC	0.526(0.476-0.591)	0.454(0.313-0.532)
LymC	0.533(0.471-0.591)	0.454(0.343-0.532)
MPV	0.484(0.437-0.553)	0.489(0.381-0.58)
NeuC	0.533(0.456-0.591)	0.463(0.378-0.58)
NLCR	0.529(0.462-0.591)	0.489(0.343-0.587)
PLTC	0.536(0.458-0.591)	0.432(0.313-0.532)
Sex	0.537(0.466-0.595)	0.505(0.381-0.587)
SOFA	0.535(0.469-0.592)	**0.381(0.236-0.453)**
WBCC	0.533(0.467-0.591)	0.471(0.355-0.556)

Table J Matthews correlation coefficients (MCC) obtained on each cohort from the best performing families of machine learning models (*LR: Logistic Regression* for *SEPSIS* and *XGB: XGBoost* for *SIRS*) after the elimination of each of the corresponding feature listed on the left. Results for the complementary models are also shown as comparison. The smaller the resulting MCC, the greater the importance of the removed feature. The most important features for the best performing models are highlighted in bold.

Text F Sensitivity Analysis: Hyperparameter Optimization A hyperparameter optimization was performed to assess the robustness of the presented results. Training data were cross-validated with an internal 5-fold cross-validation for hyperparameters tuning. The hyperparameters showing the highest MCC on the internal validation set were selected and all the training data were used to refit the optimal set of parameters before testing on the test set. The following hyperparameters were optimized:

- SVM: $kernel = [\text{"}linear\text{"}, \text{"}rbf\text{"}]$, $C = [100, 10, 1.0, 0.1, 0.01]$
- LR: $C = [100, 10, 1.0, 0.1, 0.01]$
- *Tree*: $criterion = [\text{"}gini\text{"}, \text{"}entropy\text{"}]$, $max\_depth = [2, 3, 5]$, $min\_samples\_split = [3, 5]$, $min\_samples\_leaf = [1, 2, 3, 5]$, "$classifier\_max\_features$":["$sqrt$", "$log2$", $None$], $class\_weight = [None, \text{"}balanced\text{"}]$
- RF: $n\_estimators$=[100, 250, 500], $max\_depth = [2, 3, 5]$, $max\_features$=["$sqrt$", "$log2$", $None$]
- XGB: $n\_estimators$=[100, 250, 500], $max\_depth = [2, 3, 5]$, $learning\_rate$=[0.1, 0.5, 1]

Results are very close to those observed without hyperparameter optimization and they are reported in the table below as the median MCC across the 100 MCCV runs for each model family.

Model	***SEPSIS***	***SIRS***
SVM	0.458196	0.343125
LR	0.515889	0.378864
TREE	0.457740	0.326395
RF	0.502100	0.402112
XGB	0.441304	0.447655

Table K Median Matthews correlation coefficients (MCC) obtained on the 100 Monte Carlo CV runs by performing hyperparameters optimization for each family of machine learning model.
