# Supplementary material for: Identifying prognostic factors for survival in intensive care unit patients with SIRS or sepsis by machine learning analysis on electronic health records
Source: PLOS Digit Health. 2024 Mar 15;3(3):e0000459. doi: 10.1371/journal.pdig.0000459 (PMC10942078; doi:10.1371/journal.pdig.0000459)
Supplement: Attachment — Submitted filename: reply_to_reviewers.pdf [file pdig.0000459.s003.pdf]

Article ID: **PDIG-D-23-00039**
Article title: **Identifying prognostic factors for survival in intensive care unit patients with SIRS or sepsis by machine learning analysis on electronic health records**
Journal: **PLOS Digital Health**
Authors: **Maximiliano Mollura, Davide Chicco, Alessia Paglialonga, Riccardo Barbieri**
Corresponding author: **Davide Chicco**
Corresponding author email: **davidechicco@davidechicco.it**
Reviews delivery date: **26th April 2023**
Reviews round: **first**

Response to reviewers

23rd June 2023

Dear editor Nan Liu,

Our replies to all the comments raised by the reviewers can be found below, in blue text.
In our replies below, all the line numbers and the page numbers refer to the new submitted version of the manuscript with tracked changes.
We submit our new, revised version of the manuscript in two versions: a version where tracked changes are denoted in blue text, and a clean version with only black text.
All the authors reviewed and approved the current version of the manuscript that we submit here.

We really hope our manuscript can be now accepted for publication in PLOS Digital Health.

Thanks again and best regards,

Davide Chicco
(on behalf of all the authors)

~ : ~ : ~ : ~ Reviewer #1 ~ : ~ : ~ : ~

1.0. The study by Mollura et. al., applied supervised machine-learning models to data collected from patients admitted to intensive care unit to highlight differences in clinical features for survival.
There are a number of major issues that need to be addressed:

Abstract:
1.1. "However, major differences between these two populations when estimating patients' risk after ICU admission are not fully elucidated."

Which subsequent risk is being referred to in this sentence?

Authors: We thank the Reviewer for having pointed this out. We updated the sentence as follows:
"*However, the characteristics associated with the improvement in the patient conditions during the ICU stay were not fully elucidated for each population as well as the possible differences between the two.*"

Author summary:
1.2. "However, distinguishing between a systemic inflammatory response syndrome (SIRS) and sepsis is not straightforward and the major differences related with patients' risk are not fully elucidated."
The authors indicate the inability to differentiate between SIRS and sepsis as the major concern. And this point is reiterated in the introduction.

Authors: Thank you for this comment; to address it, we have updated the author summary by keeping the focus on the study's goal. The first part of the summary now reads:
"*Systemic inflammatory response syndrome (SIRS), an exaggerated response of the body to a noxious stressor, and sepsis, an organ dysfunction caused by a dysregulated host response to infection, are two of the most critical conditions in the intensive care unit showing high patients' mortality and resulting in an important global health problem. However, the major differences leading to an improvement in the patient conditions during SIRS and sepsis are not fully elucidated. [...]*"

Why the focus on prognostic factors for survival after either SIRS or sepsis?
There is some disconnect.

Authors: We agree with the Reviewer that the justification of the study focus was not clearly presented in the original manuscript. To resolve the disconnect, we have modified the introduction to provide a more appropriate context to this study and to better clarify that the goal of this study is to understand what are the key factors leading to a better outcome for the SIRS and SEPSIS populations, as well as to highlight the major differences characterizing the two.

Introduction:
1.3. Why the distinction between patients with SIRS and sepsis?
Will it make a difference if the authors just identified the cohort as patients admitted to ICU, in general?
The aim of the study is to predict survival in ICU patients.

Authors: Thank you for bringing this up. The major reason leading to a separate analysis between SIRS and sepsis populations is due to the interest for the prognostic factors that may differ between the two populations. This study is not just focusing on the

generic survival prediction of ICU patients but on the investigation of the most important prognostic factors from blood count parameters for each population, thus providing insights regarding the variables that should be more closely monitored for each population to enable evaluation of risk and therefore prevention.

Following the Reviewer's comment we modified the introduction by highlighting this point as reported below:
"[...] *The authors found that the combinations of CRP, lymphocytes count (LymC), and platelet count (PLTC) can be used to determine the likelihood of sepsis,* ***however without exploring the association of these parameters with the patient survival for each population. This information can provide significant indications about the most important prognostic factors specifically for non-sepsis SIRS and sepsis patients*** [..]"

Methods:
1.4. The dataset did not have information on comorbid conditions at the time of ICU admission. The presence of comorbid conditions impacts on survival of ICU patients. This was not accounted in the models. Does this impact on the models?

Authors: Thank you for highlighting this aspect. The lack of information about comorbidities in this study sample is certainly an important issue and therefore a limitation of the study that deserves a deeper discussion. We do expect that model performances could benefit from more detailed information about the patients' history and that full characterization of the patient's health history would be important. We now highlight this point in the discussion section as shown below:
"[..] *Specifically, we performed the evaluation of the ability of hemogram parameters in predicting the survival of ICU patients diagnosed with SIRS or SEPSIS, using a set of parameters usually available in the patient clinical records. We used widely available features like patient sex, illness severity scores commonly measured and recorded at admission in the ICU, C-reactive protein, and blood cell count measurements.* ***Patients' comorbidities were not available in the patients' records shared by Gucyetmez et al. despite the fact that they are commonly available in an ICU setting, which represents a significant lack of information. The developed models would have certainly benefited from more information about the patient's history and this could have led to a more precise identification of differences in the prognostic factors. Therefore, future studies will focus on the extension of these analyses on more complete data including patients' comorbidities.***
The survival prediction models were developed and tested on SIRS and SEPSIS cohorts,
with better performances observed in the SEPSIS cohort. [...] "

1.5. "It is important to notice that these data were collected originally for administrative health reasons, and not for scientific purpose." The authors considered this a strength and did not highlight potential limitations of using such administrative data for a scientific purpose.

Authors: We would like to thank the Reviewer for this comment and we agree that this sentence was not properly justified. Following the Reviewer's comment we have more deeply analyzed the source of the data. Actually, for the original dataset authors did not mention whether the data are collected for administrative purposes or whether they are commonly used for clinical practice. Therefore, we decided to move this sentence in the 'General Applicability section' and adapt it as indicated below:
" *It is important to notice that we are not aware of whether these data were collected for administrative health reasons or whether they are commonly used for clinical practice, which might limit the general applicability of the results. However, the employment of data like these for scientific analyses based on computational intelligence can allow new scientific discoveries that otherwise would be impossible with traditional hospital technologies"*

1.6. Missing data and how missing data was handled was not discussed in the manuscript.

Authors: The original data shared by Gucyetmez et al. contained only 3 missing data in three separate records. We therefore simply removed the records with missing information.

1.7. Association between features and survival - how was this assessed? The sentence does not provide the details: "Association between the input features and patient survival was also explored with classical statistical approaches."

Authors: Thanks for this question. In section "Associations between features and survival", more information about the used approaches can now be found:
In particular, the following sentence describes the approaches: "*specifically, differences in numeric features between survived and deceased groups in each cohort were tested with the Mann-Whitney U test, whereas differences in categorical features were assessed with* $\chi 2$*-test [29]. Statistical significance was defined for* $p<0.005$ *as advocated by Benjamin et al. [30]*"

1.8. A study flow diagram will be needed to help explain what was done.

Authors: Thanks for this suggestion. We have updated the manuscript as suggested by the reviewer with the following flow diagram (newly added Figure 1)

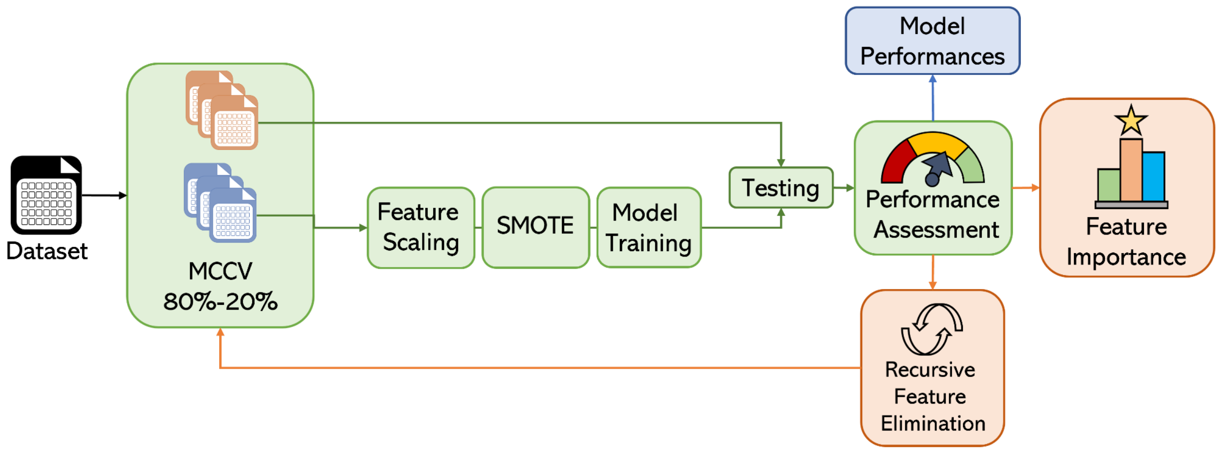

1.9. Was the data split for the 2 analyses: SIRS-related analysis using data from 816 patients and sepsis-related analysis using data from 441 patients?

Thank you for your comment. Yes, we have performed two distinct analyses on the two populations. We have revised the manuscript in order to make it clearer to the readers by adding the following sentence:
"*In this retrospective study, we developed predictive models of patient survival using machine learning algorithms and we evaluated the importance of features associated with patient survival using machine learning and biostatistics approaches for both the SIRS and SEPSIS populations separately*"

1.10. Statistically, was the study adequately powered?

Authors: We thank the reviewer for this comment. Since a statistical analysis on this dataset was already published by the original authors, we considered the dataset viable for a machine learning analysis, thus we did not perform a-priori statistical power analysis. We do recognize the importance of adequate statistical power and therefore we looked at previously published studies predicting the patient's outcome with hemogram parameters, finding that they have collected a similar amount of patients, i.e. from 150 to 1,000 patients. Therefore, based on these earlier studies we assumed the population adequate for this study. Please find below some references:

- Firat, A., Ercan, A., Mordeniz, C., & Verit Atmaca, F. F. (2023). Predictive value of hemogram parameters in malignant transformation of the endometrium in patients with different risk factors. In T. Urbanowicz (Ed.), PLOS ONE (Vol. 18, Issue 1, p. e0279224). Public Library of Science (PLoS). https://doi.org/10.1371/journal.pone.0279224

- Acar E, Demir A, Yıldırım B, Kaya MG, Gökçek K. The role of hemogram parameters and C-reactive protein in predicting mortality in COVID-19 infection. Int J Clin Pract. 2021 Jul;75(7):e14256. doi: 10.1111/ijcp.14256. Epub 2021 Apr 30. PMID: 33887100; PMCID: PMC8250321.
- Asaduzzaman MD, Romel Bhuia M, Nazmul Alam Z, Zabed Jillul Bari M, Ferdousi T. Significance of hemogram-derived ratios for predicting in-hospital mortality in COVID-19: A multicenter study. Health Sci Rep. 2022 Jun 7;5(4):e663. doi: 10.1002/hsr2.663. PMID: 35686199; PMCID: PMC9172589.
- Hsu CW, Chen NC, Huang WC, Lin HC, Tsai WC, Huang CC, Cheng BC, Tsai NW. Hemogram parameters can predict in-hospital mortality of patients with Myasthenic crisis. BMC Neurol. 2021 Oct 6;21(1):388. doi: 10.1186/s12883-021-02412-4. PMID: 34615473; PMCID: PMC8493047.
- REMAP-CAP Writing Committee for the REMAP-CAP Investigators. Effect of Antiplatelet Therapy on Survival and Organ Support–Free Days in Critically Ill Patients With COVID-19: A Randomized Clinical Trial. JAMA. 2022;327(13):1247–1259. doi:10.1001/jama.2022.2910

Dataset reference: Gucyetmez B, Atalan HK (2016) C-Reactive Protein and Hemogram Parameters for the Non-Sepsis Systemic Inflammatory Response Syndrome and Sepsis: What Do They Mean? PLOS One 11(2): e0148699. https://doi.org/10.1371/journal.pone.0148699

1.11. Can the authors clarify which model is "Decision Tree (Tree)"? Random forest and XGBoost are decision tree-based algorithms or models.

Authors: Thanks for pointing it out. In our study, we have defined “Tree” a classification model based on one single decision tree. Random Forest and XGBoost are considered Tree-based because these ensemble models were trained considering many Tree models as weak learners.

1.12. Could the authors explain why this was done and provide reference for this: "Features were standardized removing the median and dividing by the interquartile range, both estimated on the training set, and five machine learning classifiers were used to develop patient survival prediction models."

Authors: We applied feature scaling before training the machine learning models as known best-practice which helps models converging faster and more accurately. To further support this, we have added the following reference [37] to the mentioned sentence in this revised manuscript:

> Géron, A. (2017). Hands-on machine learning with scikit-learn and tensorflow: Concepts. *Tools, and Techniques to build intelligent systems*.

1.13. Is Monte Carlo stratified cross-validation a cross-validation approach or a feature importance or ranking approach?

Authors: Thanks for your question. We have used a Monte Carlo stratified cross-validation to obtain average performances of the proposed approach. We have now updated the abstract to better clarify this concept.

1.14. Will it be helpful if area-under the curve and calibration plots are provided to assess the performance of the models in addition to MCC?

Authors: Calibration curves may not be sufficiently reliable in the presence of strongly imbalanced datasets such as the one used in our study. In particular, the problem of course is that with a low incidence of the target class (and a small population) the conditional class membership probabilities will almost always be high (both observed and predicted), so the farther to the left and down we go in the diagram, the less reliable it is, and sometimes even never explored due to the difficulty of obtaining very low observed and predicted probabilities (which result in truncation of the plot).
Also, considering that we are looking at the average performance of several Monte Carlo cross-validated models, it is only possible to estimate an average calibration of the proposed approach that due to the limited number of data will certainly need some *ad hoc* optimization of both hyperparameters and output probabilities before the deployment.

Nevertheless, as per the Reviewer's request we report calibration curves for the best and worst cases for both SIRS and SEPSIS populations. Here, it can be observed how the model consistently underestimates real probabilities. In particular, for SEPSIS the bias is so consistent that it could be easily corrected by simply adding a correction factor for lower predicted probabilities (e.g. <80%). On the other hand, looking at the curve for the SIRS population, it is not possible to explore the full calibration because low probabilities cannot be observed. WIth all due respect, considering the technical limitations of the construction procedure of calibration curves with this dataset, we would prefer not to include them in the main manuscript. Nevertheless, we report this information below for your consideration and, if you deem this information is critical and should be added to the manuscript, we may include the curves in the supplementary file.

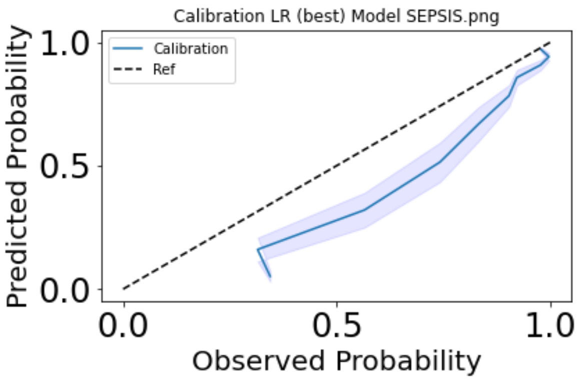

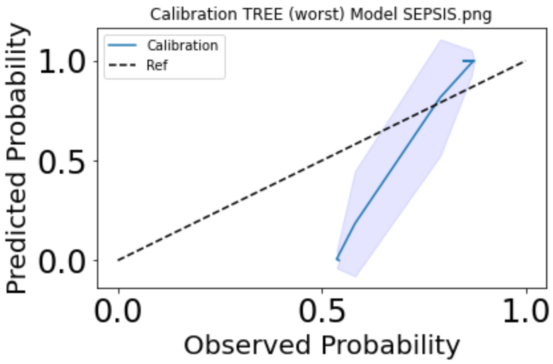

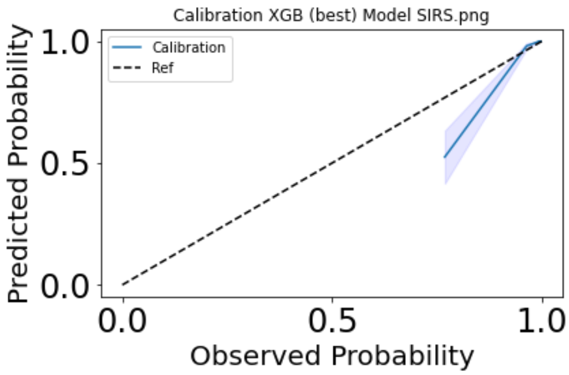

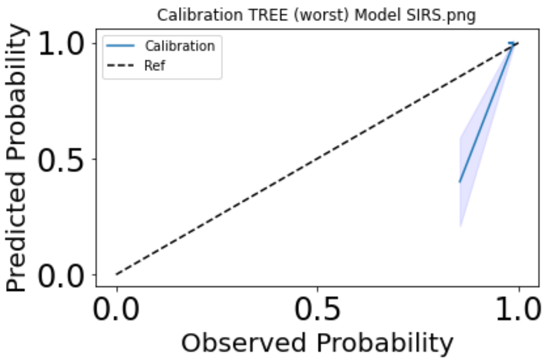

1.15. Page 6 has Fig 2 showing ROC curves - there is no mention of ROC curve in the methods section.

> Authors: Thanks for highlighting this point. We now mention the ROC and PRC curves in the methods section.

Minor concern:
1.1. The authors use the word "multivariate" a number of times in the manuscript. This should be "multivariable".

> Authors: Thanks for highlighting this mistake, we have modified the word throughout the manuscript following the reviewer’s suggestion.

~ : ~ : ~ : ~ Reviewer #2 ~ : ~ : ~ : ~

2.0 Mollura et al present in this paper a machine learning approach to predict survival of patients admitted to the ICU having systemic inflammatory response syndrome or sepsis. The authors investigate the performance of five machine learning models and subsequently estimate the importance of the input features. The paper is well written and states clearly the intentions.

> Authors: We thank the reviewer for the positive feedback and for the valuable comments, which helped us prepare an improved and clearer version of our manuscript.

However, I have some concerns in relation to how the results are generated methodologically and therefore the overall value of the work is somewhat unclear. I also have some doubts on the actual clinical relevance of the paper. I acknowledge that a prototype of a method can be valuable, even if it in its present form would not be finalized for implementation in an ICU electronic patient record system.

> Authors: Regarding the clinical relevance of this study, in this revised manuscript we more clearly justify that we propose a machine learning-based methodology to explore in multivariable settings the role of hemogram parameters as prognostic factors for the patients' survival. Indeed the aim is to use this framework to understand the most relevant factor which can indicate a potential improvement in the patients' conditions while correcting for the patient's characteristics (e.g. age, sex) and the baseline risk (APACHE II, SOFA), as such providing a basis for future investigation of patient-specific approaches to monitor, predict, and improve patient outcomes through the analysis of EHR data.

Major concerns:

2.1. I really appreciate that the authors use a correlation coefficient for evaluating model performance – and in this way take into account all elements of the confusion matrix. Nevertheless, I find it somewhat misleading that the outcome encoded as 1 is survival and not death (table 1 says that the feature 'mortality' encodes death as 1, correct me if this means that you encoded death in the outcome with 1 - in that case you can ignore the rest of this point). Using survival as target outcome is counterintuitive, since the rare event usually is the one encoded in this way. To this end I believe that the design makes AUROC, MCC and NPV informative metrics only. Performances in the supplementary tables are exceptionally good, with AUPRC values of > 95% in most cases. An AUPRC of 0.998% implies you are extremely good at predicting the positive class, but in this case the positive class is survival, which accounts for the majority of the population (~97% for the SIRS population).
This would have not been the case if death was encoded as 1. Even if this is not an error, I believe that this decision makes the quantitative interpretation of the metrics misleading. Another note is that for the metrics that are not "areas under …" it is not mentioned explicitly

what the probability threshold used for the calculation is. Calibration curves are not included at all, which makes the clinical utility of the model less clear.

Authors: We thank the Reviewer for these insightful comments and we agree that class labeling is an important aspect.
As in this study we decided to focus on the identification of the factors associated with a good prognosis of the patient instead of patient mortality, we used label 1 for survival. Given the importance of a proper weighting of both outcomes we decided to use the Matthew correlation coefficient (MCC) as the main metrics as it is invariant with respect to class labeling, thus assuring that we are not underestimating the impact of misclassifying patient's mortality. Moreover, the MCC gives the same importance to positives and negatives, so we can confirm that even if switching positive data elements with negative data elements, we would have obtained the same results in terms of MCC scores. We have now removed the 1-0 labels in the dataset description in Table 1 to avoid any misunderstandings.

Regarding the probability threshold, we have also revised the supplementary material indicating 0.5 as the threshold considered to compute the other scores: when predictions were greater or equal to 0.5, we mapped them into 1s, and when predictions were below 0.5, we mapped them into 0s.

Regarding calibration curves we have found some issues during the creation of the curve itself due to the strongly imbalanced dataset. In particular, the problem of course is that with a low incidence of one of the two classes (and a small population) the conditional class membership probabilities will almost always be high (both observed and predicted), so the farther to the left and down we go in the diagram, the less reliable it is, and sometimes even never explored due to the difficulty of obtaining very low observed and predicted probabilities (which result in truncation of the plot).
Also, considering that we are looking at the average performance of several Monte Carlo cross-validated models, it is only possible to estimate an average calibration of the proposed approach that due to the limited number of data will certainly need some *ad hoc* optimization of both hyperparameters and output probabilities before the deployment.

Nevertheless, as per Reviewer's request we report calibration curves for the best and worst cases for both SIRS and SEPSIS populations. Here, it can be observed how the model consistently underestimates the real probabilities. In particular, for SEPSIS the bias is so consistent that it could be easily corrected by simply adding a correction factor for lower predicted probabilities (e.g. <80%). On the other hand, looking at the curve for the SIRS population, it is not possible to explore the full calibration because low probabilities cannot be observed. With all due respect, considering the technical limitations of the construction procedure of calibration curves with this dataset, we would prefer not to include them in the main manuscript. However, we add this information

below for your consideration and if you deem this information is critical for the manuscript, we may include them in the supplementary file.

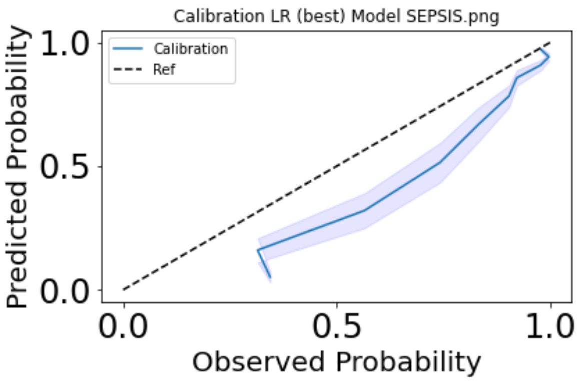

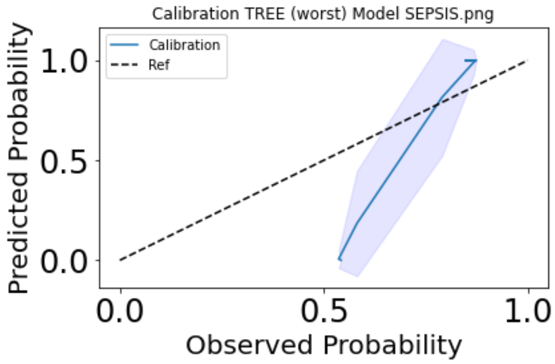

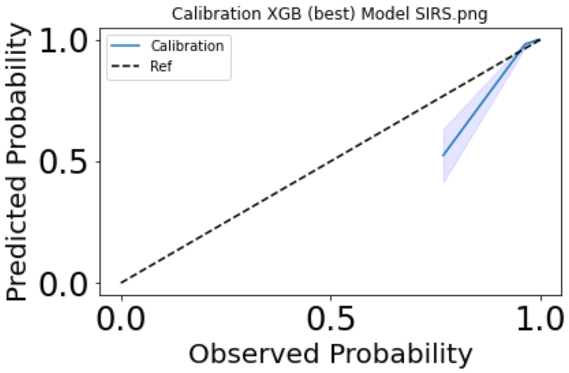

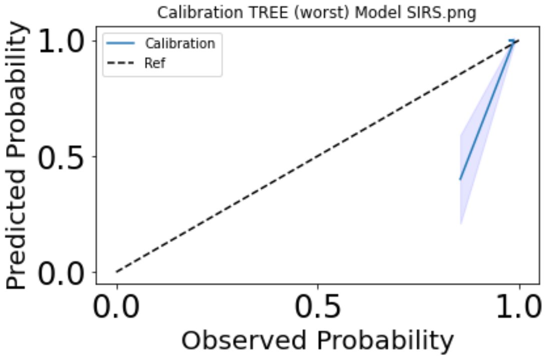

2.2. It is not stated at what timepoint the features were included and at what timepoint the survival was assessed. The authors also do not explain how multiple measurements were aggregated. This is very important in order to understand the study design. Also, no information about imputation and missing values has been included.

Authors: Thank you for your comment and for highlighting that these aspects were not clearly presented in our original manuscript. The considered data were collected from Gucyetmez and colleagues at the ICU admission and prior to any medical treatment. Aggregation and Imputation were not performed because the data were almost complete and punctual. Only 3 records showed missing data in sex or diagnosis (admission reason), and the corresponding records were removed from the study. Patient's mortality/survival was assessed at the end of their ICU stay.
We have now added this information to the manuscript in the methods section.

2.3. The authors did not provide any information about which hyperparameters were used to run the five different ML models. This is also a very important aspect of the study design.

Authors: Following the Reviewer's suggestion, we now report all the hyperparameters on the supplementary material.

2.4. I do not fully understand the way the feature importance was calculated. RFE should routinely remove the least important feature (evaluated using MCC, according to what the authors state), until a desired number of features is reached. In Results, the MCC is reported for each model with only one feature knocked out at a time. This makes the whole feature interpretation difficult to assess as most of the features are not independent or even included into one another (e.g. age into APACHE). This causes features like age, which is usually a very strong predictor of ICU survival, not important because even if explicitly removed from the feature set, it is still implicitly used for prediction by including the APACHE feature. The same argument is also valid for other features.

Authors: Thanks for this question. In this study, we are not performing Backward Feature Selection. We have selected a-priori the features that should be used by the model. In particular, we use Recursive Feature Elimination, i.e. we remove iteratively only one feature at a time to analyze the observed loss in prediction performances after training and testing again the algorithm. This approach provides an assessment of the importance of each feature in predicting patients' survival by focusing on the resulting MCC score. Therefore, a stronger reduction in MCC would imply a stronger importance of the removed variable.

2.5. It is unclear whether multiple test correction has been used to evaluate the associations between features and survival ?

Authors: Yes, we have decided to set a lower threshold for statistical analyses in order to account for multiple testing (now explicitly stated in the main manuscript in lines 84-85).

2.6. The code used for the analysis has not been provided. This is a very important aspect.

Authors: As suggested by the Reviewer, we now provide the GitHub link to our software code.

Minor concerns:

2.7. I can see that LOS-ICU and diagnosis have not been included in the feature set. While I understand the reason this has been done for LOS-ICU (leakage of information as this feature is only available after the outcome actually happens), I do not fully understand why 'diagnosis' has not been used.

Authors: In this study, we decided not to include the 'diagnosis' (i.e., the ICU admission reason, which is coded with general categories, i.e., medical, elective, or emergency surgery, as shown in Table 1, and does not include information about the individual

diseases/conditions). By excluding the feature ‘diagnosis’, we aimed to understand the importance of patients’ individual and clinical characteristics with respect to survival, with the more general goal of developing a monitoring system independent from the specific clinical pathway (and decisions) that led the patient in the ICU. Indeed we tried to avoid any possible source of bias or correlation linked with the ICU admission reason which might encode information about the patients’ characteristics that we would expect might be encoded also in the considered parameters (for example, APACHE II and SOFA scores provide a quantitative description of the patient’s conditions at the admission that would be correlated with the admission reason).

2.8. The statement on normalization is mentioned twice: “Features were standardized removing the median and dividing by the interquartile range, …” and immediately after “Features were rescaled before feeding them to the classifier by removing the median and dividing by the interquartile range …”.

Authors: Thank you for pointing this out. We have corrected the manuscript accordingly.

2.9. The feature ‘sex’ is indicated as gender in the data set section, please standardize.

Authors: Thank you for this comment, we have standardized the terms.

2.10. In my opinion, this sentence does not really belong to the dataset section : “In this retrospective study, we developed predictive models of patient survival using machine learning algorithms and we evaluated the importance of features associated with patient survival using machine learning and biostatistics approaches. “

Authors: Thank you for your suggestion. We have revised the manuscript by rearranging the manuscript structure. In particular, we have moved the Dataset section before “Materials and Methods” (now renamed as “Methods”) and the sentence mentioned by the Reviewer is now used as the opening of the “Methods” section.

2.11. The authors used violin plots. These plots have the disadvantage of showing the full tail of a distribution. In Figure 1 for example many models have MCC scores lower than 0 (it is a bit surprising to have such a wide range of MCC values) . I would suggest instead to use a boxplot, to limit the visualization to the interquartile range and outliers.

Authors: We thank the Reviewer for this suggestion. We now show boxplots in the revised manuscript.

2.12. Conclusion: while the paper has many valuable aspects and is well written, the unclear methodology and the lack of clear technical descriptions make it very hard to assess the value of the approach.

Authors: We would like to thank the Reviewer again for the feedback provided and the specific, valuable suggestions. We hope we have addressed all the Reviewers’ points and we now provide an updated version of the manuscript which aims to be clearer and more detailed.

~ : ~ : ~ : ~ : ~ : ~ : ~ : ~ : ~ : ~ : ~ : ~ : ~
