# Supplementary material for: Identifying prognostic factors for survival in intensive care unit patients with SIRS or sepsis by machine learning analysis on electronic health records
Source: PLOS Digit Health. 2024 Mar 15;3(3):e0000459. doi: 10.1371/journal.pdig.0000459 (PMC10942078; doi:10.1371/journal.pdig.0000459)
Supplement: Attachment — Submitted filename: reply_to_reviewers2.pdf [file pdig.0000459.s004.pdf]

Article ID: **PDIG-D-23-00039R1**
Article title: **Identifying prognostic factors for survival in intensive care unit patients with SIRS or sepsis by machine learning analysis on electronic health records**
Journal: **PLOS Digital Health**
Authors: **Maximiliano Mollura, Davide Chicco, Alessia Paglialonga, Riccardo Barbieri**
Corresponding author: **Davide Chicco**
Corresponding author email: **davidechicco@davidechicco.it**
Reviews delivery date: **31st July 2023**
Reviews round: **second**

Response to reviewers

19 September 2023

Dear editor Nan Liu,

Our replies to all the comments raised by the reviewer can be found below, in blue text.
In our replies below, all the line numbers and the page numbers refer to the new submitted version of the manuscript with tracked changes.
We submit our new, revised version of the manuscript in two versions: a version where tracked changes are denoted in blue text, and a clean version with only black text.
All the authors reviewed and approved the current version of the manuscript that we submit here.

We really hope our manuscript can be now accepted for publication in PLOS Digital Health.

Thanks again and best regards,

Davide Chicco
(on behalf of all the authors)

~ : ~ : ~ : ~ Editor ~ : ~ : ~ : ~

Thank you for the revision. While we see some parts of the manuscript have been improved, there are still major concerns, such as calibration and clinical relevance, among others. Please carefully address reviewers' concerns and comments, provide detailed responses, and make necessary further revisions. It is important to ensure your research results are translatable into clinical practice.

Authors: We thank both Editor and Reviewers for their valuable feedback. We do think all your suggestions allowed us to significantly improve our manuscript.

~ : ~ : ~ : ~ Reviewer #2 ~ : ~ : ~ : ~

The authors have in technical terms replied to all the points, however, calibration for example is about making a method work in practice or at least substantiating that it has that potential. The are also other points I believe are not fully clear and overall the paper is not ready for publication in my view.

2.1 - My main concerns are in particular the two aspects, model calibration and feature interpretation. I understand and agree that calibration curves in cases where the dataset is very imbalanced are often far from the actually observed probabilities. But in these cases one would expect to see mitigation with Platt scaling or isotonic regression on the probabilities. If the decision is to proceed without calibration adjustment, I would still include those calibration curves in the supplementary and specify in the Discussion this as a limitation. The model as it stands can only be used to classify two groups depending on a single decision threshold and cannot provide an individualized survival probability – and this is in my view a limiting factor. Another comment related to the revised version is that the authors did not use any hyperparameter search. As they have shown in the supplementary materials, they used the models with the default parameters as directly provided by scikit-learn. This may not be a problem per-se but I wonder if they could have obtained better performances if they had explored other model configurations. This again makes this translational value unclear. It would even be unethical to use a method in the clinic that was suboptimal, given the data, and we do not know if this is the case.

Authors: We thank the reviewer for this comment. We totally agree on all the statements about calibration. It is unfortunate that the limited number of patients did not allow us to apply both calibration and hyperparameter search. However, we also think that our results are sound provided this limitation. To further clarify this issue, we decided to run a separate analysis in order to provide a more detailed assessment of the model performances, indeed by applying a hyperparameter search as suggested by the reviewer:

-Training data are cross-validated with an internal 5-fold cross-validation for hyperparameters tuning. The hyperparameters showing the highest average MCC on the internal validation set are selected and all the training data are used to refit the model with the optimal set of parameters before testing on the test set.
-Results are very close to those observed without hyperparameter optimization and they are reported in the table reported below, where the median of the 100 Monte Carlo runs for each model family is displayed

Model	Sepsis	SIRS

SVM	0.458196	0.343125
LR	**0.515889**	0.378864
TREE	0.457740	0.326395
RF	0.502100	0.402112
XGB	0.441304	**0.447655**

The best results (in bold) are very close to those obtained with fixed parameters (MCC without Hyperparameter tuning: LR Sepsis 0.533 - XGB SIRS 0.489). Actually, the results with hyperparameter optimization are slightly lower, thus suggesting an increased risk of overfitting, likely due to the further reduction in the sample size. Of note, the hyperparameter search in the context of a Monte Carlo CV of our dataset yields different hyperparameters for each of the 100 models trained.
We have described and added this sensitivity analysis in the supplementary material to highlight the robustness of the obtained results without hyperparameter optimization and we further discussed the main limitations of our approach.

Importantly, as suggested by the reviewer, the calibration curves have now been added to the supplementary material and the related major limitations are also presented in the main text.

2.2 - The feature interpretation is still not clear to me. The response to my previous question "In particular, we use Recursive Feature Elimination, i.e. we remove iteratively only one feature at a time to analyze the observed loss in prediction performances after training and testing again the algorithm. " seems not to be correct. RFE does not iteratively remove only one feature at a time. Reference 42 used to explain the method says .. "(a) running RF to determine initial importance scores, (b) removing the bottom 3% of variables with the lowest importance scores from the data set (3% was chosen because of the high computational demands of using a lower threshold; this resulted in a total of 324 RF runs), and (c) assigning ranks to removed variables according to the order in which they were removed and their most recent importance scores (i.e., importance scores are only compared within runs, not between runs). This was performed iteratively using the reduced data set created
in step two until 3% of the "number of remaining variables" rounds to zero (i.e., no further variables could be removed)." This definition is also consistent with how sklearn (which was not used by the authors to calculate RFE in their notebook) defines it (https://scikit-learn.org/stable/modules/generated/sklearn.feature_selection.RFE.html). The method "removes one feature at a time" to my knowledge would not account for correlated variables as it is done in reference 42 with RFE.

Authors: We understand the reviewer's concerns. We do agree that the proposed name of our approach might be misleading. Therefore, we now propose our feature importance approach under a different name, and we have adapted the manuscript accordingly.

The proposed approach to assess feature importance is now referred to as “Single Feature Elimination”, as it consists in assessing the influence on the model performances of the exclusion of one feature while keeping the others. Any loss in performance can therefore be considered strictly associated with the importance of the excluded feature.

2.3 - This paper does not really propose a novel framework (as the authors state in the Discussion), and overall I think the implementation of the standard methods along with the limited clinical utility of it makes the paper somewhat incremental.

Authors: We have now updated the ‘Discussion’, ‘General considerations and applicability’ and ‘Conclusions’ to clarify the added value, the novelty, as well as the major limitations of our study.

~ : ~ : ~ : ~ : ~ : ~ : ~ : ~ : ~ : ~ : ~ : ~ : ~
