# Supplementary material for: Identifying prognostic factors for survival in intensive care unit patients with SIRS or sepsis by machine learning analysis on electronic health records
Source: PLOS Digit Health. 2024 Mar 15;3(3):e0000459. doi: 10.1371/journal.pdig.0000459 (PMC10942078; doi:10.1371/journal.pdig.0000459)
Supplement: Attachment — Submitted filename: 3rd reply to reviewers.pdf [file pdig.0000459.s005.pdf]

Article ID: **PDIG-D-23-00039R2**
Article title: **Identifying prognostic factors for survival in intensive care unit patients with SIRS or sepsis by machine learning analysis on electronic health records**
Journal: **PLOS Digital Health**
Authors: **Maximiliano Mollura, Davide Chicco, Alessia Paglialonga, Riccardo Barbieri**
Corresponding author: **Davide Chicco**
Reviews delivery date: **27th October 2023**
Reviews round: **third**

Response to reviewers

6th December 2023

Dear editor Nan Liu,

Our replies to all the comments raised by the reviewer can be found below, in blue text.
In our replies below, all the line numbers and the page numbers refer to the new submitted version of the manuscript with tracked changes.
We submit our new, revised version of the manuscript in two versions: a version where tracked changes are denoted in blue text, and a clean version with only black text.
All the authors reviewed and approved the current version of the manuscript that we submit here.

We really hope our manuscript can be now accepted for publication in PLOS Digital Health.

Thanks again and best regards,

Davide Chicco
(on behalf of all the authors)

~ : ~ : ~ : ~ Editor’s comment ~ : ~ : ~ : ~

Thank you for addressing Reviewer 2's comments. We noticed that Reviewer 1's comments were accidentally stored in the wrong place, thus you were unable to see them. Below please find Reviewer 1's comments for Revision 1 & Revision 2. Apologies for any inconvenience.

> Authors: Thanks for including reviewer’s 1 comments from our first revision. We provide here below a revised response to reviewer’s 1 comments, highlighting all the changes introduced in the manuscript during all review stages, we hope this final report will resolve the confusion due to the first incomplete round.

Authors' note for reviewer 1: We thank the reviewer for his/her first feedback based on our previous version. We answer these important points here below (1st revision), and we also address the reviewer's new comments (2nd revision) further below. We hope that, despite this inconvenience, we were able to provide a clear portrayal of all the important improvements deriving from the reviewer's feedback.

~ : ~ : ~ : ~ Reviewer #1 on 1st revision ~ : ~ : ~ : ~

(1.1.0) Thank you to Mollura and colleagues for the effort in addressing the initial questions and suggestions - very much appreciated.
I am, however, unsure about the clinical relevance and how this study could possibly impact patient care. My main concerns are:

(1.1.1) From the response, the data being used for this study is from the study: https://doi.org/10.1371/journal.pone.0148699 This data was collected over a decade ago. Patient characteristics and disease profiles change based on advances in medical practice. Over the past decade, there has been significant advances and patient profiles have changed. What is the impact of this on the study findings? Risk predictions models, for example, are revised/updated to deal which such changes.

> Authors: We thank the reviewer for pointing it out. We do agree that the considered data, collected over a decade ago, might be quite old. However, these precious data were collected in a similar temporal period of some of the most popular public ICU databases like MIMIC-III [1] and eICU [2], which are commonly used for retrospective analysis and predictive modelling in literature. In addition, the definition of sepsis is still strongly debated due to the large spectrum of phenotypes associated with it. Gucyetmez and colleagues [3] defined sepsis in this according to its second definition, which was strongly correlated with SIRS thus making the two very difficult to distinguish. This is at the core of our investigation, aiming at identifying the key element from hemogram parameters predicting patient's survival that might differ between these two (very similar) populations.
>
> References:

> [1] Johnson, A., Pollard, T., Shen, L. et al. MIMIC-III, a freely accessible critical care database. Sci Data 3, 160035 (2016). https://doi.org/10.1038/sdata.2016.35
> [2] The eICU Collaborative Research Database, a freely available multi-center database for critical care research. Pollard TJ, Johnson AEW, Raffa JD, Celi LA, Mark RG and Badawi O. Scientific Data (2018). DOI: https://doi.org/10.1038/sdata.2018.178.

[3] Gucyetmez B, Atalan HK (2016) C-Reactive Protein and Hemogram Parameters for the Non-Sepsis Systemic Inflammatory Response Syndrome and Sepsis: What Do They Mean? PLOS One 11(2): e0148699. https://doi.org/10.1371/journal.pone.0148699

(1.1.2) The research idea and concept is great, but is the data being used the most appropriate to address this research question?

Authors: Although the considered data present some limitations, both in terms of numerosity and lack of general patient's conditions, we do consider this dataset particularly interesting. Indeed, blood samples used to derive hemogram parameters had been taken at ICU admission prior to any medical treatment, which we consider a major strength of Gucyetmez and colleagues' [3] dataset, and this condition is unlikely in public datasets. Therefore, any successive investigations will benefit from a baseline analysis run on this dataset.

[3] Gucyetmez B, Atalan HK (2016) C-Reactive Protein and Hemogram Parameters for the Non-Sepsis Systemic Inflammatory Response Syndrome and Sepsis: What Do They Mean? PLOS One 11(2): e0148699. https://doi.org/10.1371/journal.pone.0148699

(1.1.3) Calibration curves are highlighting significant imbalanced data. The models are consistently underestimating real probabilities. Could this be discussed/highlighted in the discussion section? How will this possibly impact on clinical implications?

Authors: This is a very important point, raised by both reviewers. Following this suggestion, we have already added all the details of model calibration in the Supplementary material, and we now clearly present this issue in the discussion section together with additional comments about the clinical implications as suggested by the reviewers (see page 9 at lines 185-190).
In particular, as shown in our study, the calibration of the best-performing model is not far from being optimal, and this issue can be addressed by the model's output re-calibration on a validation population. This approach would also account for a possible covariate shift due to change in patient characteristics, without the need for development of a new model. Therefore, we do consider that the relationships between the available variables, both intra- and inter-population, can be considered as a reliable multivariable comparison of the major factor predicting survival for both SIRS and sepsis patients.

~ : ~ : ~ : ~ Reviewer #1 on 2nd revision ~ : ~ : ~ : ~

(1.2.0) My review was not made available to the authors in the decision letter, hence these have not been addressed.
Additionally, based on authors' response to concerns from the other reviewer, I am not convinced about the clinical importance and how the findings can be translational at this stage.

Major concerns:
(1.2.1) Small sample size

(1.2.2) No external validation of the models that have been developed. The current model after further sensitivity analysis is overfitting.

(1.2.3) Authors choosing to change labels of methods supposedly used in the analysis without evidence to support the label.

Many thanks for the opportunity to review this.

Kind regards

Authors: We thank the reviewer for this second feedback based on the response to the other reviewer. We have reported above the responses to your points in your first review and, at the same time, we address your new comments here below:

(1.2.1) - In this last version of the manuscript we now extensively discuss the limitation related with small sample size. Despite recognising the importance of sample size, we do consider this dataset particularly useful to define the baseline response of hemogram parameters in ICU patients. This important aspect, strictly related to the translational utility of the methods considered, is now pointed out even more strongly in the current revised version (see revised discussions and limitations, and also response to your first review).

(1.2.2) - Lack of external validation stems from sample size limitation. We make the case that our current results provide a very important outcome as related to the study of the association between the considered variables in these two populations. Any attempt to move toward external validation studies on other popular datasets would have implied the consideration of several additional confoundings, thus moving the focus away from the specific aims of this study. For example, the considered data have been collected at admission and before initiation of any treatment protocol, a condition quite difficult to be met when considering most public databases.

(1.2.2) - Regarding overfitting, we have carefully applied the presented model to avoid as much as possible technical overfitting. Indeed we included the sensitivity analysis at a second stage after the other reviewer suggestion during second round of revision where we gave the following answer:

> *"Actually, the results with hyperparameter optimization are slightly lower, thus suggesting an increased risk of overfitting, likely due to the further reduction in the sample size. Of note, the hyperparameter search in the context of a Monte Carlo CV of our dataset yields different hyperparameters for each of the 100 models trained."*

In light of this answer, it should be noted that the mentioned increased risk of overfitting was referring to the models trained with the hyperparameter optimization step, which requires an internal validation set, thus further reducing the population size. However, apart from the separate sensitivity analysis, the proposed models in the study are not trained with hyperparameter optimization. In any case, the fact that sensitivity analysis shows similar results further corroborates the Monte Carlo procedure as a reliable estimating model approach independently from the specific set of hyperparameters.

(1.2.3) Regarding labelling swap, we do highlight that in this study we decided to focus on the identification of the factors associated with a good prognosis of the patient instead of patient mortality, we used label 1 for survival. In any case, in order to avoid underestimation of mortality, we decided to use the Matthews correlation coefficient (MCC) as main metrics due to its invariance with respect to class labeling. In the second revision, we have removed the 1-0 labels in the dataset description in Table 1 to avoid any misunderstandings.

~ : ~ : ~ : ~ ~ : ~ : ~ : ~ ~ : ~ : ~ : ~ ~ : ~ : ~ : ~

Reviewers' comments:

Reviewer's Responses to Questions

Reviewer #2: Unfortunately some sections were modified but not marked in blue. Nevertheless I think the authors addressed all the comments I had. I would add in limitations that the SFE method is not fully informative when features are highly interdependent – since the contribution of a feature that is very important may still result as not important during SFE because of its contribution through other covariates that depend on it. With this additional remark in the Discussion I believe the manuscript is ready for publication.

We thank Reviewer #2 for her/his comments and suggestions. We have now modified the ‘General Considerations and Applicability’ section following reviewer suggestion as reported here below:
“ It is important to notice that the SFE method presents some limitations when features are highly interdependent, since the contribution of a feature that is very important may still be underestimated due to the effect of other covariates that depend on it.”
